# MRI assessment of myocardial global function by tei index - a comparison to echocardiographic tei index

**DOI:** 10.1186/1532-429X-13-S1-P333

**Published:** 2011-02-02

**Authors:** Hazel D Rovno, Harold Litt, Victor Ferrari

**Affiliations:** 1 Hospital of the University of Pennsylvania, Philadelphia, PA, USA

## Introduction

The Tei index is an echocardiographic measure of global ventricular function, calculated as ((isovolumic contraction time) + (isovolumic relaxation time)) / (ejection time). This may be calculated as ((time of atrioventricular valve closure) - (time of arterial valve closure)) / (time of arterial valve closure). Used as a measure of systolic as well as diastolic function, the Tei index has been validated for use in the left heart and right heart, in normal patients and those with a variety of pathologies. Measures of systolic and diastolic function by MRI, with few exceptions, are not directly comparable to echocardiographic measures.

## Purpose

To measure an MRI-derived Tei index, and compare it to the echo based Tei index.

## Methods

Retrospective, focused on the left ventricle. Medical record review disclosed 40 patients, both normal and with a variety of pathologies, with MRI and echo examinations suitable for review. Patients had an echocardiogram with appropriate M-mode or Doppler and had MRI with an LVOT view or stacked cine view for evaluating mitral and aortic closure. MRI images were preferred which included aortic and mitral valves on the same slice. Next preference was given to stacked images acquired over the same cardiac cycles. For echocardiogram assessment, M-mode valve images were preferred; if not available, Doppler waveforms were used. We assessed mitral valve closure time, aortic ejection time, Tei index, and systolic left ventricular function by MRI and by echocardiography.

## Results

In general correlation between MRI measures and corresponding echo measures was not as good as expected, possibly because the time between echocardiogram and MRI was variable, and it was not possible in this small retrospective study to control for altered clinical status (e.g., volume status, blood pressure changes). Even for well-validated measures such as ejection fraction, the MRI-echo correlation in our sample was r^2^=0.42. Mitral closure time was less well correlated between MRI and echo (r^2^ = 0.23), and while there was a general trend in the appropriate direction, MRI-echo Tei index correlation approached zero.

## Conclusions

Initial attempts to make MRI measurements that are more directly comparable to standard echocardiographic measures were not successful. Time between examinations may have played a role, and a prospective study may be necessary to achieve better correlation.

